# Gelam Honey Inhibits the Production of Proinflammatory, Mediators NO, PGE_2_, TNF-****α****, and IL-6 in Carrageenan-Induced Acute Paw Edema in Rats

**DOI:** 10.1155/2012/109636

**Published:** 2012-07-01

**Authors:** Saba Zuhair Hussein, Kamaruddin Mohd Yusoff, Suzana Makpol, Yasmin Anum Mohd Yusof

**Affiliations:** ^1^Department of Biochemistry, Faculty of Medicine, University Kebangsaan Malaysia, Jalan Raja Muda Abdul Aziz, 50300 Kuala Lumpur, Malaysia; ^2^Department of Molecular Biology and Genetics, Faculty of Arts and Science, Canik Basari University, 34083 Samsun, Turkey

## Abstract

Natural honey is well known for its therapeutic value and has been used in traditional medicine of different cultures throughout the world. The aim of this study was to investigate the anti-inflammatory effect of Malaysian Gelam honey in inflammation-induced rats. Paw edema was induced by a subplantar injection of 1% carrageenan into the rat right hind paw. Rats were treated with the nonsteroidal anti-inflammatory drug (NSAID) Indomethacin (10 mg/kg, p.o.) or Gelam honey at different doses (1 or 2 g/kg, p.o.). The increase in footpad thickness was considered to be edema, which was measured using a dial caliper. Plasma and paw tissue were collected to analyze the production of inflammatory mediators, such as NO, PGE_2_, TNF-**α**, and IL-6, as well as iNOS and COX-2. The results showed that Gelam honey could reduce edema in a dose-dependent fashion in inflamed rat paws, decrease the production of NO, PGE_2_, TNF-**α**, and IL-6 in plasma, and suppress the expression of iNOS, COX-2, TNF-**α**, and IL-6 in paw tissue. Oral pretreatment of Gelam honey at 2 g/kg of body weight at two time points (1 and 7 days) showed a significantly decreased production of proinflammatory cytokines, which was similar to the effect of the anti-inflammatory drug Indomethacin (NSAID), both in plasma and tissue. Thus, our results suggest that Gelam honey has anti-inflammatory effects by reducing the rat paw edema size and inhibiting the production of proinflammatory mediators. Gelam honey is potentially useful for treating inflammatory conditions.

## 1. Introduction

Inflammation is a complex biological response of the body to cell damage and vascularized tissue, which can be classified as either acute or chronic depending on the time of onset [1]. Acute inflammation is the body's primary response to injurious stimuli, and some of the body's responses are characterized by pain, heat, redness, swelling, and loss of function [2, 3]. During an inflammatory response, several proinflammatory mediators are released, including interleukin 1 (IL-1), IL-6, IL-12, tumor necrosis factor (TNF), and interferon (INF-*γ*) as well as cyclooxygenase-2 (COX-2) and inducible nitric oxide synthase (iNOS) [4]. These cytokines play major roles in the initiation and amplification of inflammatory processes [5]. Nitric oxide (NO), a free radical generated by inducible nitric oxide synthase (iNOS), can act as a defense and regulatory molecule with homoeostatic activities. However, it can also be pathogenic when it is produced excessively [6]. 

From ancient times until now, several natural products and their derived formulations have been used in therapeutic applications for inflammatory disorders and related diseases [7, 8]. Curcumin was effective at preventing acute liver damage indicated by the reduced expression of proinflammatory cytokines and the deactivation of NF*κ*B in an animal model study [9]. Ginger extract has a potent anti-inflammatory property by inhibiting the production of proinflammatory mediators, NO and PGE_2_, in LPS-stimulated macrophage RAW 264.7 cells [10].

Honey is a natural product of honey bees, that is, derived from floral nectars and other plant secretions [11]. Honey is rich in carbohydrates, proteins, vitamins, trace elements, enzymes, and phenolic compounds [12]. Since archaic times, honey has been used as an ingredient of traditional medicine because of its dietary and curative properties. Scientific studies over the past ten years have shown that honey possesses various important biological properties, such as wound healing [13, 14] and antibacterial [15], antioxidant [16–18], antitumor [19, 20], and anti-inflammatory effects [21–23].

In Malaysia, there are several types of honey, including Tualang, Nenas, Coconut, and Gelam. Among these, Tualang and Gelam honeys are well known in Malaysia for their potential health benefits, such as antioxidant and anti-inflammatory activities [24–26]. Mohamed et al. [27] have shown that Tualang honey contains highly phenolic compounds that possess relatively good antioxidant activity. In an animal model, a topical dressing of Tualang honey showed a positive effect for treating full-thickness burn wounds [28]. We have previously reported that Gelam honey has antioxidative and radical scavenging activities, which are mainly attributed to its phenolic content [29]. Other biological activities of Gelam honey that have been reported are wound healing [30], anti-inflammatory effects [25], and antioxidant activity [26].

In the present study, we investigated the anti-inflammatory activity of Gelam honey in short and long durations of treatments in rats that were induced with paw edema.

## 2. Materials and Methods

### 2.1. Chemicals

All of the chemicals and reagents used were of analytical grade. Indomethacin and *λ*-Carrageenan were obtained from Sigma Chemicals Co. (USA). The anesthetized KTX mixture (Ketamine, Tiletamine/Zolazapam and Xylazine) was supplied by Universiti Kebangsaan Malaysia Animal Ethics Committee (UKMAEC), Laboratory Animal Resource Unit, (Malaysia). Skim milk (Sunlac, Malaysia), Trizma Base, protease inhibitor cocktail, urea, thiourea, CHAPS detergent, sodium dodecyl sulfate (SDS), and Tween 20 were all supplied by Sigma Chemical Co. (Germany). The membrane polyvinylidene fluoride (PVDF) was purchased from GE Healthcare (USA), and chemiluminescence was supplied by Perkin Elmer (USA).

### 2.2. Honey Sample

Malaysian monofloral Gelam honey is produced by *Apis mellifera*, and the major nectar and pollen collected by the bees is from the plant *Melaleuca cajuputi *Powell, which is known locally as the “Gelam tree.” It was provided by the National Apiary, Department of Agriculture, Batu Pahat, Johor, Malaysia. The Gelam honey was packed in plastic bottles and was sent to SINAGAMA, Malaysian Nuclear Agency for a sterilization process using a Cobalt-60 source (Model JS10000). The irradiation process was conducted at a dose of 25 kGy [31]. The honey was stored at 4°C in the dark until the analysis was performed. HPLC analysis of Gelam honey for the determination of phenolic compounds were reported in Hussein et al. [29].

### 2.3. Animals

A total of 84 male Sprague-Dawley rats weighing (200–300) g were obtained from the Laboratory Animals Resource Unit, Faculty of Medicine, The National University of Malaysia. The rats were housed in individual cages under standard conditions (Temperature at 22 ± 2°C, 12 hr light/dark cycle) and were fed with laboratory chow and water *ad libitum*. Experiments were performed during the light phase of the cycle. Prior to their use, they were allowed one week for acclimatization within the work area environment. All of the experimental protocols used in this study were approved by the Animal Ethics Committee of the National University of Malaysia, Malaysia (date of approval 17th March 2010: pp/BIOK.2010/Yasmin).

### 2.4. Carrageenan-Induced Paw Edema in Rats

The anti-inflammatory effect of honey was evaluated by a subplantar injection of carrageenan into the footpad of the right hind paw of rats, as described previously by Winter et al. [32]. Two models were employed in this study, with each model consisting of seven groups (*n* = 6 rats for each group). The first model represents rats that were pretreated with Gelam honey for 1 day, while the second model represents rats that were pretreated with Gelam honey for 7 days. In both of the models, the rats were pretreated orally with honey once daily at two different doses (1 or 2 g/kg of body weight). The negative control received an equivalent volume of vehicle (distilled water), and the positive control group received the nonsteroidal anti-inflammatory drug (NSAID) Indomethacin (IND, 10 mg/kg of body weight) [33]. One hour after the last day of administration of Gelam honey, vehicle or Indomethacin, the rats in both of the models were injected subcutaneously onto the plantar surface of the right hind paw with (0.2 mL/paw) 1% carrageenan in saline [25].  Table 1 showed the details of the treatment groups. After the carrageenan injection, the paw thickness was measured at several time points (0–6, 12 and 24 hr) using a Dial Caliper (0–150 mm/0.02 mm, Mitutoyo, Japan). The paw thickness was determined at 0 hr (*C*_0_: paw thickness before carrageenan injection) and at 1, 2, 3, 4, 5, 6, 12, and 24 hr after carrageenan injection (*C*_*t*_). The percentages of inhibition compared to negative controls (inflammation group) were calculated according to the following formula:

(1) %Inhibition =[(Ct−C0)control−(Ct−C0)treated group](Ct−C0)control×100

Three groups (*n* = 6) in each model acted as normal controls that received distilled water and honey at two different doses (1 or 2 g/kg of body weight) orally. The normal control groups were not induced with inflammation by carrageenan.

### 2.5. Measurement of Paw Edema

Paw thickness was used as a measurement of inflammation-induced edema [34]. Briefly, the dorsoventral thickness of each hind paw was measured using a caliper placed at the border of the phalanges and metatarsals. The measurement was taken when each edge of the caliper was just touching the dorsal and ventral surface of the hind paw (the caliper was not squeezed onto the hind paw). Data are expressed as the mean paw thickness ± S.E.M.

### 2.6. Preparation of Blood Plasma Samples

Twenty-four hours after carrageenan injection, the rats were anesthetized with a KTX mixture (0.1 mL/100 g of body weight), and the blood was collected from the orbital sinus in heparinized tubes. The blood was centrifuged at 1500 ×g for 10 min (4°C); the plasma was aliquoted and stored at −20°C until use.

### 2.7. Preparation of Tissue Samples

Rat paw tissue segments measuring 0.5 cm were cut and washed in normal saline several times. They were snap frozen in liquid nitrogen and stored at −80°C until they were used for Western blot and RT-PCR analyses.

### 2.8. Measurement of Nitric Oxide (NO) in Plasma

Nitric oxide production was measured using the QuantiChrom Nitric Oxide Assay Kit (Bioassay Systems, USA), which estimates NO from the concentrations of nitrate and nitrite according to the Griess method. Initially, sodium nitrite standard curve was prepared (ranges from 0–100 *μ*M). Deproteination of the plasma was accomplished by mixing 150 *μ*L with 8 *μ*L ZnSO_4_ in a 1.5 mL tube, followed by 8 *μ*L of NaOH, vortexing, and centrifuging for 10 min at 14,000 rpm. Then, 100 *μ*L of clear supernatant was transferred to a clean tube. Prior to starting the reaction, the working reagent for all of the samples and standards was prepared. An amount of 100 *μ*L of each sample and standard were added to 200 *μ*L of the working reagent and incubated for 10 min at 60°C. The reaction tubes were then centrifuged and 250 *μ*L of each reaction was transferred to separate wells in a 96-well plate. The optical density was read at 540 nm. The total nitrite concentrations were determined by comparisons to the sodium nitrite standard curve, and the results were expressed as (*μ*M) nitrite.

### 2.9. Measurement of Prostaglandin (PGE_2_) in Plasma

Production of PGE_2_ was measured by the PGE_2_ Express Enzyme-Immuno Assay- (EIA-) Monoclonal kit (Cayman Chemical, Ann Arbor, MI, USA). The analysis was performed according to the manufacturers' guidelines. Briefly, 50 *μ*L of plasma was used in 96-well plates coated with goat anti-mouse IgG antibodies; 50 *μ*L of tracer and 50 *μ*L of the specific antibody were added to each well. The plates were incubated for 1 hr at room temperature. All of the wells were washed five times with washing buffer followed by adding 200 *μ*L of Ellman's reagent (acetylthiocholine and 5,5′-dithio-bis-[2-nitrobenzoic acid]) to each well, and the plates were incubated in the dark at room temperature for 60–90 min. This procedure allowed the bound enzyme tracer to react with Ellman's reagent, which yielded a yellow solution that can be measured photometrically with a microplate reader at 420 nm.

### 2.10. Determination of TNF-*α* and IL-6 in Plasma

(TNF-*α*) levels were estimated in the rat's plasma using rat-specific TNF-*α* sandwich ELISA (IBL International GmbH, Hamburg, Germany) according to the manufacturer's protocol. Briefly, a 96-microwell plate coated with a polyclonal antibody to rat TNF-*α* was used. The microwell plate was washed with wash buffer and was allowed to sit in the wells for approximately 10–15 seconds before aspiration. After the last wash step, the microwell plate was tapped onto an absorbent pad or paper towel to remove excess wash buffer. Standard wells include 100 *μ*L of serial diluted concentration of rat TNF-*α*: 2500, 1250, 625, 312.5, 156.3, 78.1, and 39.1 pg/mL. Sample wells include 50 *μ*L of sample diluents with 50 *μ*L plasma samples. An amount of 50 *μ*L of biotin-conjugate was added to all of the wells. The plate was covered with adhesive film and was incubated for 2 hr at room temperature, on a shaker. All of the wells were washed four times with washing buffer, followed by the addition of 100 *μ*L of streptavidin-HRP, and the plate was covered with adhesive film and was incubated for 1 hr at room temperature, on a shaker. All of the wells were washed four times with washing buffer; finally, 100 *μ*L of TMB substrate solution was added to all of the wells, and the plate was incubated in the dark for 10 min at room temperature. The enzyme reaction was stopped quickly by pipetting 100 *μ*L of stop solution into each well, and the plate was read immediately using a microplate reader (VersaMax-Tunable Microplate Reader, USA) at 450 nm.

IL-6 was measured in plasma samples using a rat-specific IL-6 ELISA kit (IBL International GmbH, Hamburg, Germany) according to the manufacturer's protocol. The method is similar to the determination of TNF*-*α** ELISA with the exception that a microwell plate coated with a monoclonal antibody of IL-6 was used instead.

### 2.11. Real-Time Polymerase Chain Reaction (RT-PCR)

The paw tissue was dissected, snap-frozen in liquid nitrogen, and stored at −80°C until analysis. Total RNA was extracted from the tissue samples using an RNeasy Mini kit (QIAGEN, USA) in an RNase-free environment, according to the manufacturer's instructions. Single-stranded cDNA was synthesized from the total RNA using an iScript cDNA synthesis kit (BIO-RAD, USA). Real-time PCR for the proinflammatory mediator's genes or housekeeping gene glyceraldehyde-3-phosphate dehydrogenase (GAPDH; the sequence-specific primer pairs were designed using the National Centre for Biotechnology Information, NCBI, website; see Table 2) was performed using SYBER-green detection (BIO-RAD, USA) in an iQ5 real-time cycler machine (BIO-RAD, USA). The cycling conditions were as follows: initial denaturation at 95°C for 3 min and amplification for 40 cycles (95°C for 10 sec for the denaturation, 56°C for 30 sec for the annealing and extension). The relative amount of gene expression, normalized to the internal control *GAPDH, *was calculated according to the following formula:

(2) relative expression values =##gt##2(Ct GAPDH−Ct target gene),

where *C*_*t*_ = the cycle at threshold level.

### 2.12. Preparation of Cytosolic Extracts

To determine the protein expressions, protein was extracted from paw tissue according to Drew et al. [35], with some modifications. A 0.5 cm paw segment was ground with liquid nitrogen until it turned into powder. An amount of 200 *μ*L of 40 mM Tris/HCl, pH 7.4, containing 3% DTT and protease inhibitor cocktail, was added followed by brief sonication. The homogenate was centrifuged at 15,000 rcf for 15 min at 4°C. The soluble proteins in the supernatant were collected (solution A). The pellet was redissolved in thiourea rehydration buffer (7 M Urea, 2 M Thiourea, 40 mM DTT and 4% CHAPS) and sonicated until the pellet was totally dissolved. The mixture was centrifuged at 15,000 rcf for 15 min at 4°C, and the supernatant was collected (solution B). Both of the solutions, A and B, were pooled, and the total protein content was measured according to the Bradford assay [36].

### 2.13. Western Blot Analysis

A total of 30–50 *μ*g of protein extract was applied to 8–15% SDS-polyacrylamide gels and then transferred to Hybond-P (PVDF) membrane. The membranes were blocked in 5% skim milk in TPBS solution (0.2% Tween 20 in PBS) for 1 hr. The membranes were incubated overnight at 4°C with specific primary antibodies for the interest protein: anti-COX-2 (1 : 200 dilution; Santa Cruz, USA), anti-iNOS (1 : 1000 dilution; Abcam, USA), anti-IL-6 (1 : 500 dilution; Abcam, USA), and anti-TNF-*α* (1 : 1000 dilution; Abcam, USA). The membrane was rinsed three times with TPBS solution for 5 min each. Thereafter, the membrane was incubated for 1 hr in the shaker with secondary antibody dissolved in TPBS at a dilution of 1 : 3000. The membrane was rinsed with TPBS solution for 5 min, which was repeated three times. The membrane was incubated with 1 mL of chemiluminescence substrate for 5 min; the bands were visualized by Gel Documentation (Alpha Inno Tech, USA).

### 2.14. Statistical Analysis

The results were expressed as the mean ± S.E.M. Data were analyzed with one-way ANOVA using SPSS version 16.0 software, and the differences at level *P* < 0.05 were considered to be statistically significant.

## 3. Results

### 3.1. Effect of Honey on Carrageenan-Induced Paw Edema in Rats

**Figure 1:**
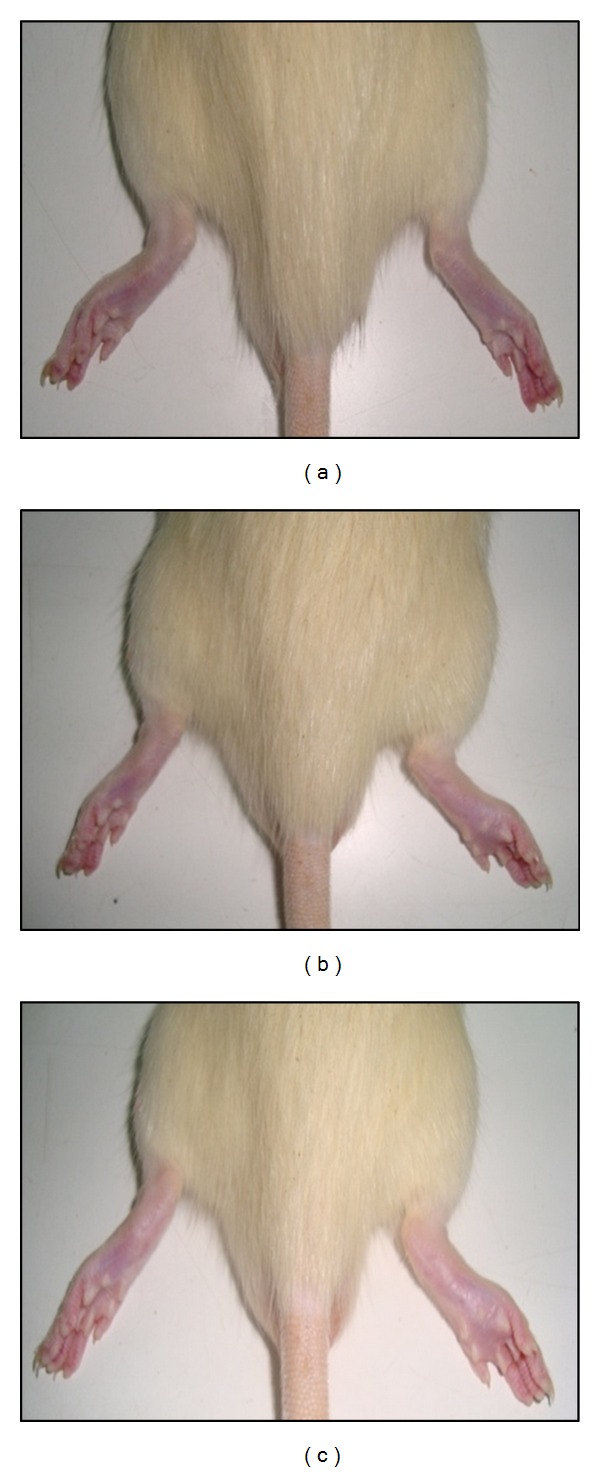
Representative photographs of rats' right hind paw (a) before (b) after 5 hr and (c) after 24 hr of carrageenan-induced paw edema inflammation.

**Figure 2:**
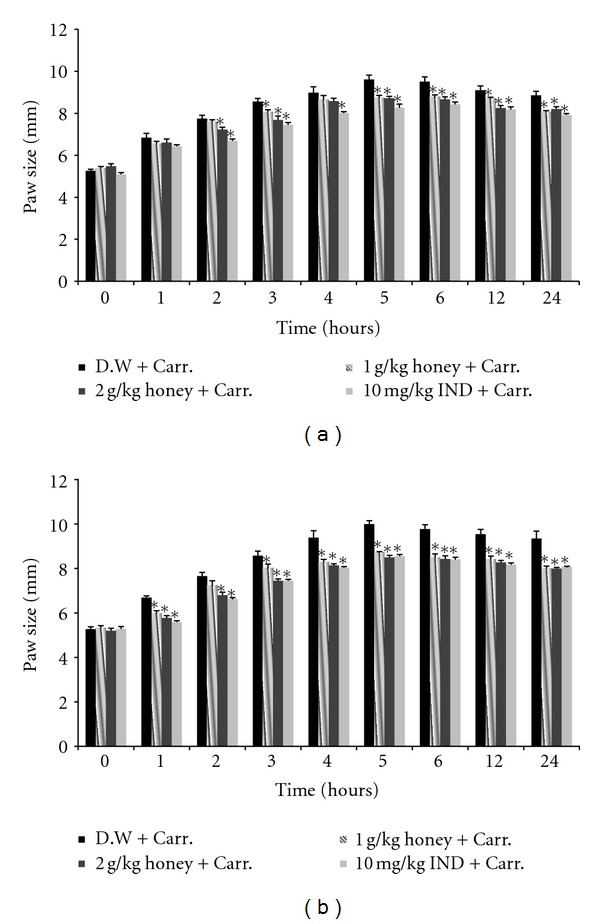
The effect of Gelam honey on the paw size in carrageenan-induced paw edema in rats. Rats were pretreated orally with Gelam honey (1 or 2 g/kg of body weight) for (a) 1 day or (b) 7 days before carrageenan injection. D.W.: distilled water, Carr: Carrageenan, IND: Indomethacin. Data are presented as the mean ± S.E.M. (*n* = 6). *Significantly (*P* < 0.05) different compared to the control (D.W. + Carr).

The subplantar injection of carrageenan in both treatment models led to a time-dependent increase in the paw size, as shown in Figure 1, which reached a maximum at 5 hr and remained elevated thereafter for 24 hr. Pretreatment of Gelam honey for 1 or 7 days at either concentration (1 or 2 g/kg of body weight) significantly (*P* < 0.05) reduced the paw edema formation in a dose-dependent manner, as shown in Figure 2. Gelam honey at 2 g/kg of body weight for 1 day decreased the paw size significantly in 2 hr, while the same dose of honey treated for 7 days significantly decreased the paw size at 1 hr after the carrageenan injection compared with the control (inflammation group). At 1 hr after the carrageenan injection, Gelam honey treated for 7 days at 1 or 2 g/kg of body weight resulted in a greater inhibition of the edema (54.23% and 59.86%, resp.) when compared with a 1-day treatment (26.58 and 29.11%, resp.).

### 3.2. The Effect of Honey on Nitric Oxide (NO) Levels in Plasma

**Figure 3:**
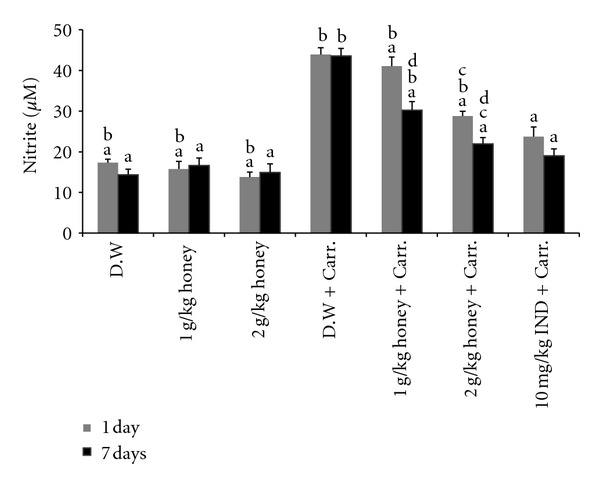
The effect of Gelam honey on NO production. Rats were pretreated orally with Gelam honey (1 or 2 g/kg of body weight) for 1 or 7 days before inflammation, as induced by carrageenan injection. D.W.: distilled water, Carr: Carrageenan, IND: Indomethacin. Data were presented as the mean ± S.E.M. (*n* = 6). ^a^ *P* < 0.05 compared to the inflammation group (D.W. + Carr.). ^b^ *P* < 0.05 compared to the Indomethacin group (10 mg IND/kg of body weight + Carr.). ^c^ *P* < 0.05 compared between different honey doses at the same time point, 1 day or 7 days. ^d^ *P* < 0.05 the same honey dose, compared at different time points, 1 day and 7 days.

Our results showed that the NO levels in the plasma of inflammation-induced rats increased significantly compared with those of the control rats (not induced with inflammation, see Figure 3). However, oral feeding with Gelam honey at either dose (1 or 2 g/kg of body weight) significantly reduced the NO levels in carrageenan-induced inflammation in rats. The effect of 2 g/kg of body weight of honey was almost similar to the NSAID Indomethacin (10 mg/kg of body weight), as shown in Figure 3. Both doses of Gelam honey inhibited the NO production dose-dependently in both pretreatment models (1 and 7 days), but the inhibition effect was greater when honey was treated for 7 days.

### 3.3. Effect of Honey on the Prostaglandin (PGE_2_) Level in Plasma

**Figure 4:**
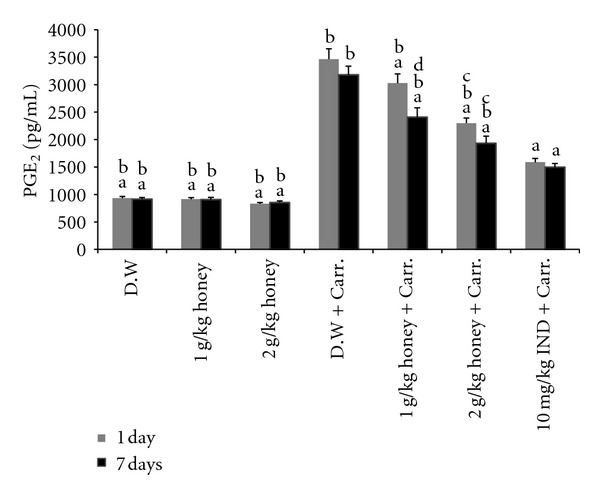
The effect of Gelam honey on PGE_2_ production. Rats were pretreated orally with Gelam honey (1 or 2 g/kg of body weight) for 1 or 7 days before inflammation, as induced by carrageenan injection. D.W.: distilled water, Carr: Carrageenan, IND: Indomethacin. Data are presented as the mean ± S.E.M. (*n* = 6). ^a^ *P* < 0.05 compared to the inflammation group (D.W. + Carr.). ^b^ *P* < 0.05 compared to the Indomethacin group (10 mg IND/kg of body weight + Carr.). ^c^ *P* < 0.05 compared between different honey doses at the same time point, 1 day or 7 days. ^d^ *P* < 0.05 the same honey dose, compared between different time points, 1 day and 7 days.

The acute inflammation by carrageenan injection induced a dramatic and significant increase in PGE_2_ production compared with the control (distilled water) (Figure 4). Gelam honey at either dose (1 or 2 g/kg of body weight) significantly inhibited PGE_2_ production in both the 1- and 7-day models, with pretreatment of 2 g/kg of body weight of Gelam honey having a greater effect on reducing the PGE_2_ production.

### 3.4. Effect of Honey on the TNF-*α* and IL-6 Levels in Plasma

**Figure 5:**
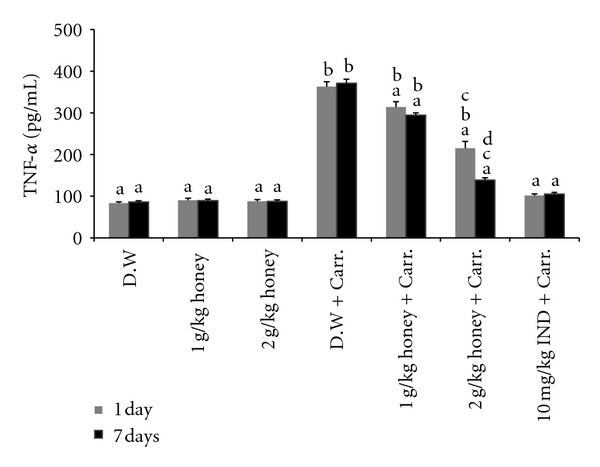
The effect of Gelam honey on TNF-*α* production. Rats were pretreated orally with Gelam honey (1 or 2 g/kg of body weight) for 1 or 7 days before inflammation, as induced by carrageenan injection. D.W.: distilled water, Carr: Carrageenan, IND: Indomethacin. Data are presented as the mean ± S.E.M. (*n* = 6). ^a^ *P* < 0.05 compared to the inflammation group (D.W. + Carr.). ^b^ *P* < 0.05 compared to the Indomethacin group (10 mg IND/kg of body weight + Carr.). ^c^ *P* < 0.05 compared between different honey doses at the same time point, 1 day or 7 days. ^d^ *P* < 0.05 the same honey dose, compared at different time points, 1 day and 7 days.

**Figure 6:**
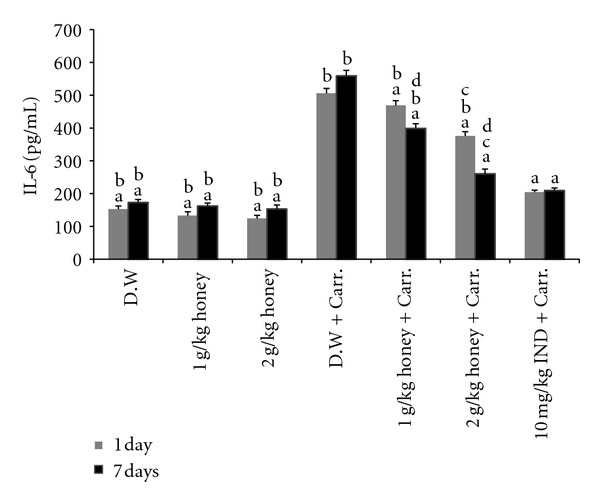
The effect of Gelam honey on IL-6 production. Rats were pretreated orally with Gelam honey (1 or 2 g/kg of body weight) for 1 or 7 days before inflammation, as induced by carrageenan injection. D.W.: distilled water, Carr: carrageenan, IND: Indomethacin. Data are presented as the mean ± S.E.M. (*n* = 6). ^a^ *P* < 0.05 compared to the inflammation group (D.W. + Carr.). ^b^ *P* < 0.05 compared to the Indomethacin group (10 mg IND/kg of body weight + Carr.). ^c^ *P* < 0.05 compared between different honey doses at the same time point, 1 day or 7 days. ^d^ *P* < 0.05 the same honey dose, compared between different time points, 1 day and 7 days.

The *in vivo* anti-inflammatory activity of Gelam honey was also monitored by evaluating the levels of TNF-*α* and IL-6 by ELISA. As shown in Figures 5 and 6, TNF-*α* and IL-6 levels increased significantly in plasma after carrageenan-induced inflammation compared to those of the control. However, pretreatment with either dose of Gelam honey (1 or 2 g/kg of body weight) decreased the production of TNF-*α* and IL-6 in plasma, in both the 1- and 7-day models, while 2 g/kg of body weight of Gelam honey decreased the production of TNF-*α* and IL-6 more than the lower dose (1 g/kg of body weight) did. Interestingly, pretreatment with Gelam honey at 2 g/kg of body weight for 7 days significantly inhibited the production of TNF-*α* and IL-6, which was comparable to the effect of the anti-inflammatory drug (NSAID) Indomethacin (10 mg/kg of body weight).

### 3.5. The Effect of Honey on the Expression of iNOS, COX-2, TNF-*α*, and IL-6 Genes and Proteins in Paw Tissue

**Figure 7:**
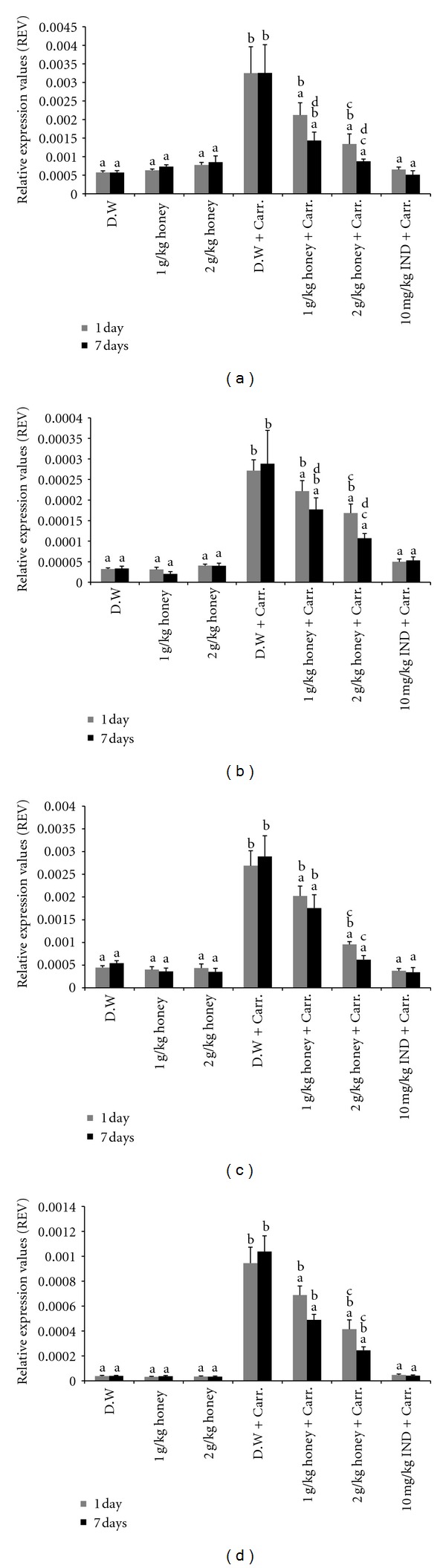
The effect of Gelam honey on the gene expression of (a) iNOS, (b) COX-2, (c) TNF-*α*, and (d) IL-6 in paw tissue. Rats were pretreated orally with Gelam honey (1 or 2 g/kg of body weight) for 1 or 7 days before inflammation, as induced by carrageenan injection. D.W.: distilled water, Carr: carrageenan, IND: Indomethacin. Data are presented as the mean ± S.E.M. (*n* = 6). ^a^ *P* < 0.05 compared to the inflammation group (D.W. + Carr.). ^b^ *P* < 0.05 compared to the Indomethacin group (10 mg IND/kg of body weight + Carr.). ^c^ *P* < 0.05 compared between different honey doses at the same time point, 1 day or 7 days. ^d^ *P* < 0.05 the same honey dose, compared between different time points, 1 day and 7 days.

An acute inflammatory response is characterized by the expression of a number of proinflammatory mediator's genes and proteins. Therefore, the *in vivo* anti-inflammatory activity of honey was monitored by evaluating the gene and protein expression levels of inflammatory-related enzymes (iNOS and COX-2) and some proinflammatory cytokines (TNF-*α* and IL-6), using RT-PCR and Western blot analysis. As shown from our results, carrageenan injection led to markedly elevated levels of these proinflammatory mediator genes and proteins in the inflamed paws of rats. Remarkably, pretreatment with Gelam honey (1 or 2 g/kg of body weight) suppressed the gene expression of (mention all here) significantly in rat paw tissue (Figure 7). Interestingly, the gene expressions of these mediators were significantly attenuated by 2 g/kg of body weight of Gelam honey in both the 1- and 7-day models.

**Figure 8:**
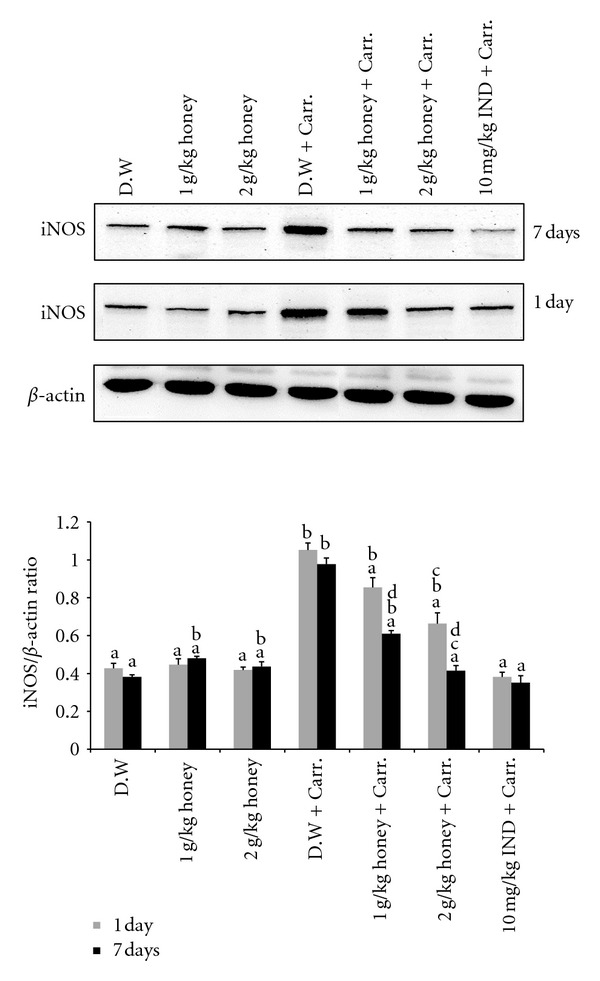
The effect of Gelam honey on the expression of iNOS protein in paw tissue. Rats were pretreated orally with Gelam honey (1 or 2 g/kg of body weight) for 1 or 7 days before inflammation was induced by carrageenan injection. D.W.: distilled water, Carr: carrageenan, IND: Indomethacin. Data are presented as the mean ± S.E.M. (*n* = 6). ^a^ *P* < 0.05 compared to the inflammation group (D.W. + Carr.). ^b^ *P* < 0.05 compared to the Indomethacin group (10 mg IND/kg of body weight + Carr.). ^c^ *P* < 0.05 compared between different honey doses at the same time point, 1 day or 7 days. ^d^ *P* < 0.05 the same honey dose, compared at different time points, 1 day and 7 days.

**Figure 9:**
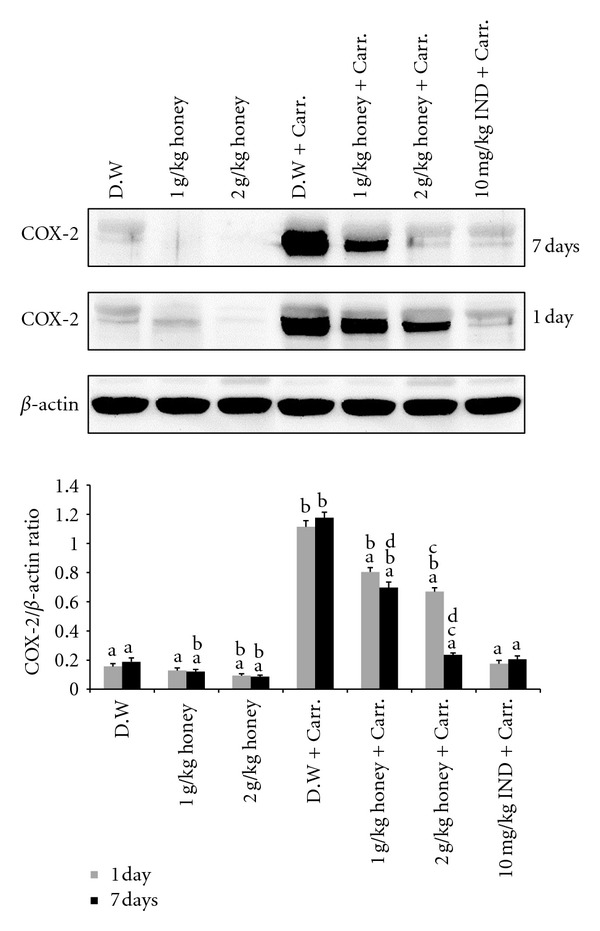
The effect of Gelam honey on the expression of the COX-2 protein in paw tissue. Rats were pretreated orally with Gelam honey (1 or 2 g/kg of body weight) for 1 or 7 days before inflammation was induced by carrageenan injection. D.W.: distilled water, Carr: carrageenan, IND: Indomethacin. Data are presented as the mean ± S.E.M. (*n* = 6). ^a^ *P* < 0.05 compared to the inflammation group (D.W. + Carr.). ^b^ *P* < 0.05 compared to the Indomethacin group (10 mg IND/kg of body weight + Carr.). ^c^ *P* < 0.05 compared between different honey doses at the same time point, 1 day or 7 days. ^d^ *P* < 0.05 the same honey dose, compared at different time points, 1 day and 7 days.

**Figure 10:**
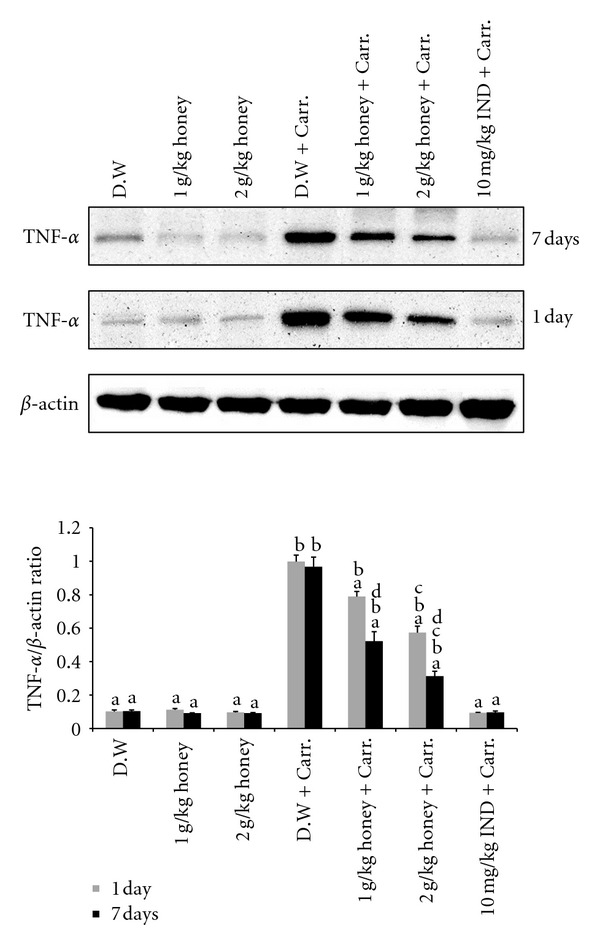
The effect of Gelam honey on the expression of TNF-*α* protein in paw tissue. Rats were pretreated orally with Gelam honey (1 or 2 g/kg of body weight) for 1 or 7 days before inflammation was induced by carrageenan injection. D.W.: distilled water, Carr: carrageenan, IND: Indomethacin. Data are presented as the mean ± S.E.M. (*n* = 6). ^a^ *P* < 0.05 compared to the inflammation group (D.W. + Carr.). ^b^ *P* < 0.05 compared to the Indomethacin group (10 mg IND/kg of body weight + Carr.). ^c^ *P* < 0.05 compared between different honey doses at the same time point, 1 day or 7 days. ^d^ *P* < 0.05 the same honey dose, compared at different time points, 1 day and 7 days.

**Figure 11:**
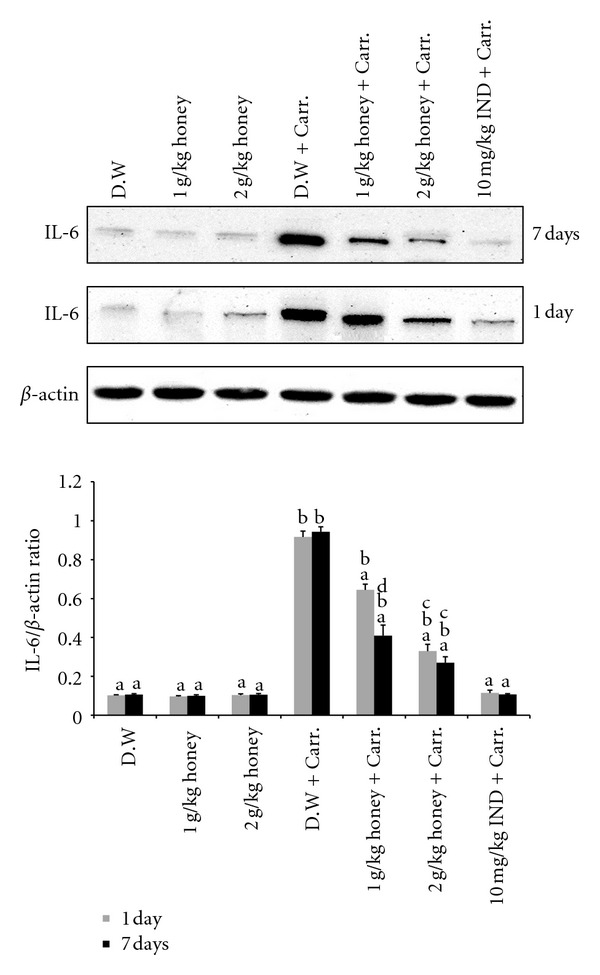
The effect of Gelam honey on the expression of IL-6 protein in paw tissue. Rats were pretreated orally with Gelam honey (1 or 2 g/kg of body weight) for 1 or 7 days before inflammation was induced by carrageenan injection. D.W.: distilled water, Carr: carrageenan, IND: Indomethacin. Data are presented as the mean ± S.E.M. (*n* = 6). ^a^ *P* < 0.05 compared to the inflammation group (D.W. + Carr.). ^b^ *P* < 0.05 compared to the Indomethacin group (10 mg IND/kg of body weight + Carr.). ^c^ *P* < 0.05 compared between different honey doses at the same time point, 1 day or 7 days. ^d^ *P* < 0.05 the same honey dose, compared at different time points, 1 day and 7 days.

Similar patterns were seen with the protein expression of these mediators; the Western blot analysis showed that 1 or 2 g/kg of body weight of Gelam honey inhibited the protein expression of iNOS and COX-2 as well as IL-6 and TNF-*α* in rat paw tissue. Pretreatment with a high concentration of Gelam honey (2 g/kg of body weight) for 7 days had a larger reduction effect on protein expression (Figures 8, 9, 10, and 11).

## 4. Discussion

Inflammation is the first response of the immune system to infection or irritation; inflammation is mediated by cytokines such as TNF-*α*, IL-1*β*, IL-2, IL-6, and PGE_2_ [37, 38]. Inhibitors of these cytokines, such as NSAID Indomethacin, Aspirin, and Naproxen, currently are the choice of anti-inflammatory agents. However, drugs are not free of side effects, which pose potential harm in the risk of other unwanted diseases.

The search for natural products with anti-inflammatory activity has increased markedly in recent years [39]. Some of these natural products include strawberry, loquat, mulberry, and bitter melon juice extracts, which decrease the secretion of proinflammatory cytokines IL-6 and IL-1*β* as well as upregulate the secretion of the anti-inflammatory cytokine IL-10 in LPS-stimulated-macrophage cells. In an *in vivo *study, curcumin and ginger extract (*Zerumbone*) attenuated inflammation through the inhibition of paw edema induced by carrageenan in an animal model [40–42].

Honey is one of the oldest known dietary medicines. It is a natural product that is synthesized by honey bees and that contains a variety of dietary nutrients that are useful for good health in humans [43]. It has been used for the treatment of respiratory, urinary, and gastrointestinal diseases [44]. Honey also promotes wound and skin ulcer healing, reduces skin inflammation, edema and exudation, diminishes scar size, and stimulates tissue regeneration [45, 46].

Our study focused on the anti-inflammatory activity of local Malaysian Gelam honey in an acute inflammation animal model. Edema is one of the fundamental actions of acute inflammation and is an essential parameter to be considered when evaluating compounds with potential anti-inflammatory activity [47, 48]. The anti-inflammatory activity of Gelam honey is clearly observed in carrageenan-induced rat paw edema, with a maximal effect 5 hr after carrageenan injection, in both 1- and 7-day pretreatment models. Kassim et al. [25] also noted a similar observation in which the subplantar injection of carrageenan led to a time-dependent increase in the paw volume, which peaked at 6 hr; however, Gelam honey reduced the edema. The oral administration of Gelam honey significantly inhibited the paw edema size at 2 and 3 hr after carrageenan injection in a 1-day pretreatment model, but in a 7-day pretreatment model, a reduction in edema started 1 hr after carrageenan injection, showing that a long duration of honey treatment provided better protection (Figure 2). The anti-inflammatory effect of 2 g/kg of body weight of Gelam honey that was observed in this study was similar to the anti-inflammatory effect of the drug Indomethacin (10 mg/kg of body weight), a well-known NSAID and a cyclooxygenase (COX-1 and 2) inhibitor. Perhaps the action of Gelam honey could be mediated by inhibiting COX-1 or 2 because the carrageenan inflammatory model basically reflects the actions of prostaglandins [33].

The development of edema induced by carrageenan is biphasic: the first phase occurs within 1 hr of carrageenan inflammation and is attributed to the release of the neurotransmitter molecules histamine and serotonin. The second phase (over 1 hr) is mediated by an increased release of prostaglandins in the inflammatory area, and the continuity between the two phases is provided by kinins [41, 49].

In the prostaglandin (PGE_2_) biosynthesis pathway, (COX-2) is the key enzyme that catalyzes the conversion of arachidonic acid to PGE_2_ [50]. PGE_2_ is a very important mediator of all types of inflammation and is responsible for increased prostaglandin production in inflamed tissue [51]. Moreover, injection with carrageenan into the rat paw induced the liberation of bradykinin, which later induces the biosynthesis of prostaglandin and other autacoids that are responsible for the formation of the inflammatory exudates [52]. Gelam honey has been reported to reduce PGE_2_ in HT29 colon cancer cells [53]. Our results indicated that both doses of Gelam honey (1 and 2 g/kg of body weight) significantly inhibited the production of PGE_2_ in the plasma of both models (1- and 7-day pretreatments), and this finding was confirmed by our observation in the paw tissue through the inhibition of the COX-2 gene and protein expression. This scenario could in part be responsible for some of anti-inflammatory properties of Gelam honey. An *in vivo *study reported that Gelam honey and its extract have anti-inflammatory effects by reducing the inflammatory mediators NO and PGE_2_ in rat paw tissue [25].

Nitric oxide (NO) is another important proinflammatory molecule that is released in an acute and chronic inflammatory response and is related to exudation and cellular chemotaxis [54]. NO is synthesized from L-arginine by nitric oxide synthase (NOS) [55] and the inducible NO synthase (NOS2 or iNOS) [56]. The inducible NO synthase (iNOS) is upregulated in response to inflammatory and proinflammatory mediators, and their products can influence many aspects of the inflammatory cascade [25]. The L-arginine-NO pathway has been proposed to play an important role in the carrageenan-induced inflammatory response [57]. NO levels have been shown to increase in the paw edema exudates that are induced by carrageenan in animal models [25, 58]. Our present results are in agreement with those previous studies that showed that carrageenan-induced inflammation in rats increased the production of NO in plasma and the expression of iNOS in paw tissue. We have also shown that Gelam honey at two different concentrations (1 and 2 g/kg of body weight) significantly decreased the levels of NO in plasma, and the reduction was a consequence of the inhibition of iNOS, the key enzyme responsible for NO production under pathological conditions. This finding indicated that perhaps Gelam honey elicits an anti-inflammatory response by inhibiting the iNOS enzyme.

Phenolic compounds have been reported to be beneficial in the treatment of chronic inflammatory diseases that are associated with the overproduction of NO and have a major role in the inhibition of PGE_2_ in inflamed tissue [25, 59]. Terra et al. [60] reported that the anti-inflammatory activity of grape seeds correlates positively with its radical-scavenging activity and its total phenolic content. Previous studies have shown that Gelam honey is high in phenolic compounds and radical scavenging activity [24, 25, 29], and it is highly likely that phenolic compounds in Gelam honey contribute to the anti-inflammatory activity [29].

Tumor necrosis factor-*α* (TNF-*α*) is a major mediator in inflammatory responses, inducing innate immune responses by activating T cells and macrophages and stimulating the secretion of other inflammatory cytokines [61, 62]. TNF-*α* has been shown to be one of the proinflammatory mediators of a carrageenan-induced inflammatory reaction and was able to induce a further release of kinins and leukotrienes, with a possible role in the maintenance of a long-lasting nociceptive response [63]. In this study, we found that Gelam honey at two different doses (1 and 2 g/kg of body weight) reduced the TNF-*α* level in plasma and its expression in paw tissue significantly after carrageenan injection, but the 2 g/kg of body weight dose decreased the TNF-*α* level significantly more than the 1 g/kg of body weight dose in both 1- and 7-day pretreatment models. As with PGE_2_ and NO, we noted that pretreatment with a higher dose of Gelam honey for 7 days significantly decreased the TNF-*α* level, similar to the NSAID Indomethacin (10 mg/kg of body weight).

Interleukin-6 (IL-6) is a cytokine that is produced during inflammation and that plays an important role in host defenses to invasive infection. IL-6 is a multifunction cytokine that is produced by a wide range of cells, usually at sites of tissue inflammation [64, 65]. IL-6 stimulates the production of most acute phase proteins in inflammatory reactions.

Changes in acute phase proteins reflect the presence and intensity of inflammation, and they have long been used as a clinical guide to diagnosis and management [66]. In our study, we found that Gelam honey at either dose (1 or 2 g/kg of body weight) decreased significantly the IL-6 levels in plasma and their expression in paw tissue after carrageenan injection, with the dose of 2 g/kg of body weight having greater effects compared to the lower dose. Similarly with the results observed with plasma levels of PGE_2_, NO, and TNF-*α*, pretreatment with the higher dose of Gelam honey for 7 days decreased the IL-6 levels, similar to Indomethacin (10 mg/kg of body weight). This finding has great potential for advocating that natural honey produces the same activity effect as the NSAID Indomethacin.

To our knowledge, no studies have reported on the effect of honey on the production of TNF-*α* and IL-6 in the plasma of an acute inflammatory model. Previous studies have reported the inhibition effect of different traditional medicine treatment on the levels of TNF-*α* and IL-6. Studies by Chun et al. [67] showed that *Moutan Cortex*, a traditional Korean herb, inhibited the levels of NO, PGE_2_, TNF-*α*, IL-1*β*, and IL-6 as well as the expression of iNOS and COX-2 in LPS-activated macrophage RAW 264.7 cells, while Huang et al. [62] reported that treatment with ethanolic extract of *Cardiospermum halicacabum, *a well-known plant used in Chinese medicine, inhibited the TNF-*α* and NO levels in serum of carrageenan-induced paw edema in mice. Furthermore, Li et al. [68] found that pretreatment with luteolin, a flavonoid that is widely distributed in plants, reduced the expression of iNOS and COX-2 proteins in LPS-induced acute lung injury in mice.

In conclusion, our results demonstrated that Gelam honey has a potent *in vivo* anti-inflammatory effect in an acute inflammation model system. Gelam honey can inhibit the production of NO, PGE_2_, TNF-*α*, and IL-6 in plasma, which corresponds to a reduction in the expression of the corresponding genes and proteins in paw tissue and to edema formation. It is possible that the mechanism of Gelam honey was associated with the inhibition of COX-2 and iNOS, which results in suppressed levels of proinflammatory mediators such as NO, PGE_2_, TNF-*α*, and IL-6. Gelam honey can be considered to be a powerful regulator of inflammation and a potential therapeutic agent against a number of diseases.

## Figures and Tables

**Table 1:** Rat treatment groups.

Groups^∗^	Treatment
1, 8	(Control) Distilled water
2, 9	(Control) Gelam honey (1 g/kg body weight)
3, 10	(Control) Gelam honey (2 g/kg body weight)
4, 11	(Inflammation) Distilled water + Carrageenan
5, 12	(Inflammation) Gelam honey (1 g/kg of body weight) + Carrageenan
6, 13	(Inflammation) Gelam honey (2 g/kg of body weight) + Carrageenan
7, 14	(Inflammation) Indomethacin (10 mg/kg of body weight) + Carrageenan

^∗^Groups 1 to 7 represent rats that were pretreated for 1 day, and groups 8 to 14 represent rats that were pretreated for 7 days.

**Table 2:** Sequences of primers and the sizes of the products.

Primer	Direction	Sequences (5^′^ to 3^′^)	Product size (bp)
GAPDH	Forward	TCAAGAAGGTGGTGAAGCAG	111
	Reverse	AGGTGGAAGAATGGGAGTTG	
COX-2	Forward	CCAAACCAGCAGGCTCATACT	183
	Reverse	AGCGGATGCCAGTGATAGAGT	
iNOS	Forward	ACCAAACTGTGTGCCTGGAGGT	174
	Reverse	TGTGCGTCTCTTCCGTGGCAAA	
TNF-*α*	Forward	TGCTCAGAAACACACGAGACGC	184
	Reverse	TTCAGCAGCCTTGTGAGCCAGA	
IL-6	Forward	TGCTCTGGTCTTCTGGAGTTCCG	182
	Reverse	AGGAGAGCATTGGAAGTTGGGGT	
